# Small renal masses

**DOI:** 10.1186/1470-7330-15-S1-O12

**Published:** 2015-10-02

**Authors:** Hersh Chandarana

**Affiliations:** 1 Department of Radiology, New York University School of Medicine, New York 10016, USA

## 

Small renal masses are increasingly diagnosed incidentally. This results in management dilemma because at histopathology significant numbers of small renal masses are either benign tumors such as angiomyolipoma (AML) or oncocytoma, or are neoplasms with relatively indolent behavior [1]. Surgical treatments such as partial and total nephrectomy although provide excellent oncologic control is associated with development and worsening of renal insufficiency and associated cardiovascular morbidity [2]. Therefore, ability to non-invasively investigate renal tumor histopathology and aggressiveness can guide treatment decision and lower treatment cost.

Within this paradigm, the role of radiologist and imaging is evolving from traditional role of identifying renal lesion and detecting enhancement, to predicting aggressiveness and biology of the tumor as well as providing operative guidance. MR imaging can play a very important role not only as a problem solving tool in traditional sense by detecting subtle enhancement and macroscopic and microscopic fat, but can provide deeper insight into tumor biology. Number of key observations highlighting the role of MR in evaluation of renal masses is as listed below:

## 1. Differentiating benign renal masses from malignant tumors

- There is some controversy regarding the role of signal loss on opposed phase chemical shift imaging in discriminating AML from RCC [3,4].

- Lipid poor AML tend to have uniform low T2 signal and uniform enhancement without evidence for necrosis [5,6].

- There is overlap in the morphologic features of Oncocytoma and RCC on conventional imaging [7]. Furthermore segmental enhancement inversion is noted in oncocytoma as well as other renal neoplasms [8].

## 2. Histologic subtyping RCC

- Papillary subtype of RCC usually have low T2 signal and are hypovascular when compared to clear cell RCC. Furthermore, clear cell subtype have heterogeneous T2 signal and demonstrate heterogeneous hypervascularity [9].

- Chromophobe subtype is difficult to differentiate from clear cell RCC on the basis of enhancement. However, advance diffusion and perfusion MR techniques have shown some promise [10].

## 3. Predicting tumor aggressiveness/outcome

- Cystic RCC with less than 25% solid enhancing component tend to be less aggressive than solid RCC [11].

- High stage clear cell RCC tend to me more heterogeneous with different texture compared to low stage RCC on Apparent diffusion coefficient (ADC) map [12].

- High grade clear cell RCC tend to have lower ADC compared to low grade clear cell RCC [13].
